# The role of neutrophil response in lung damage and post-tuberculosis lung disease: a translational narrative review

**DOI:** 10.3389/fimmu.2025.1528074

**Published:** 2025-03-07

**Authors:** Ana Paula Santos, Luciana Silva Rodrigues, Nils Rother, Fernanda Carvalho de Queiroz Mello, Cecile Magis-Escurra

**Affiliations:** ^1^ Pulmonary Diseases Department, Pedro Ernesto University Hospital, State University of Rio de Janeiro, Rio de Janeiro, Brazil; ^2^ Thoracic Diseases Institute, Federal University of Rio de Janeiro, Rio de Janeiro, Brazil; ^3^ Department of Respiratory Diseases-TB Expert Center, Radboud University Medical Center, Nijmegen, Netherlands; ^4^ Department of Pathology and Laboratories, Medical Sciences Faculty, State University of Rio de Janeiro, Rio de Janeiro, Brazil; ^5^ Department of Nephrology, Radboud University Medical Center, Nijmegen, Netherlands

**Keywords:** pulmonary tuberculosis, post-tuberculosis lung disease, lung damage, neutrophil, neutrophil elastase, neutrophil extracellular traps, matrix metalloproteinases

## Abstract

It is estimated that more than 150 million individuals alive in 2020 had survived tuberculosis (TB). A portion of this large population continues to experience chronic respiratory abnormalities, with or without symptoms, due to previous active pulmonary TB. This condition known as Post-TB Lung Disease (PTLD), involves a complex interaction between pathogen, host and environmental factors. These interactions are believed to drive a hyperinflammatory process in the lungs during active TB, resulting in tissue damage, which may lead to radiological sequelae, impaired pulmonary function, clinical symptoms, such as cough, dyspnea, hemoptysis, and respiratory infections. Such complications impose significant health, financial, and social burdens, which remain poorly understood and inadequately addressed by health care systems. Given the heterogeneity of immune cells and their products infiltrating the airways and the lung parenchyma during acute and chronic inflammation caused by *Mycobacterium tuberculosis* infection, it is evident that TB immunopathology is multifactorial. Among the various components involved, neutrophils have recently emerged as critical contributors to the deleterious immune response against TB, leading to severe pulmonary damage. In this translational narrative review, we aim to summarize the role of neutrophils and their primary products - proteases (such as elastase), matrix metalloproteinases and neutrophils extracellular traps (NETs) - in pulmonary TB. We highlight new concepts and emerging evidence of neutrophil involvement during the active disease, translating these insights from “bench to bedside” to facilitate dialogue between fundamental researchers and clinical practitioners. Additionally, we present potential targets for future treatment strategies that could mitigate or even prevent PTLD.

## 1 Introduction

*Mycobacterium tuberculosis* (Mtb), the etiological agent of human tuberculosis (TB), remains one of the world’s deadliest pathogens. Approximately 10 million people suffer from the disease each year, and, despite being a preventable and curable disease, 1.25 million people die from it annually (1). Around 85% of all active TB cases manifest as pulmonary disease (2), which not only sustains bacillary transmission but is also linked to Post-TB Lung Disease (PTLD), defined as “evidence of chronic respiratory abnormality, with or without symptoms, attributable at least in part to previous (pulmonary) tuberculosis” (3). Although a recent modeling study estimated that over 150 million individuals alive in 2020 had survived TB (4), the true global burden of PTLD remains unknown. Its clinical consequences, however, are evident, manifesting as chronic respiratory symptoms, impaired pulmonary function, and radiological sequelae, even after microbiological cure (5).

The complex interplay between pathogen, host, and environmental factors is believed to contribute to the heterogeneous nature of pulmonary TB in individuals who either fail to eliminate the bacilli after inhalation or contain them in a latent state within the granuloma (6). In patients who are unable to control mycobacteria replication and develop active disease, the virulence of Mtb plays a significant role in lung impairment (7). Additionally, environmental factors such as smoking, exposure to biofuels, and other pollutants are associated with long-term lung disease in TB survivors (8, 9). From the host perspective, several factors may influence lung damage during pulmonary TB and the development of PTLD, including innate and adaptive immunity, timing of TB diagnosis, nonadherence to anti-TB treatment, genetic polymorphisms, and hyperinflammation (6, 10, 11).

As reviewed and summarized by several authors (12–17), from the first moment of Mtb invasion into the lungs, the host initiates an innate immune response, particularly in the distal airways, represented by specialized phagocytic and antigen-presenting cells, such as alveolar macrophages and dendritic cells. These cells promote the recruitment and activation of neutrophils, monocytes, and finally the activation of protective adaptive immune response mainly with both CD4^+^ and CD8^+^ T lymphocytes, but also B cells. Further interactions between immune system and airway epithelial cells, fibroblasts, and the extracellular matrix, are mediated by a complex signaling network of biomarkers such as cytokines, chemokines, and peptidases, and culminate in granuloma formation, the central structure of mycobacterial pathogenesis. Granulomas are organized structures derived from host immune cells that surround infecting Mtb. Initially considered solely a host-protective response, containing and walling off the mycobacteria within it, the granuloma can also be beneficial to the mycobacteria, facilitating their expansion (18). Tuberculous granuloma can take many shapes and forms, including fibrotic granulomas, calcified granulomas, and suppurative granulomas (necrotic core). However, the most classic during active TB are associated with the formation of caseous necrosis, where the central region of the granuloma undergoes necrotic cell death, leading to the formation of a core of cell debris that frequently have a soft, cheese-like consistency termed caseum (18). This process may perpetuate lung inflammation and tissue destruction, leading to fibrosis and airway remodeling (19, 20).

Given the heterogeneity of immune cells and their products infiltrating the airways and lung parenchyma during acute and chronic inflammation triggered by Mtb infection, it is now well established that TB immunopathology is multifactorial. However, among all participants, neutrophils have recently emerged as key players, not only involved in early events of Mtb infection, but also contributing to the deleterious immune response to active TB disease, contributing significantly to severe lung damage (21–27).

Neutrophils, or polymorphonuclear (PMN) leucocytes, are a diverse cell population whose combined activity influences the degree of inflammation and disease outcomes during TB. Their biological properties can contribute to both protective and harmful immune responses (28), impacting TB immunopathogenesis. This translational narrative review aims to summarize the role of neutrophils in pulmonary TB and present new concepts and evidence regarding their effector mechanisms during active disease, effectively translating findings from “bench to bedside”. Our goal is to enhance the connection between fundamental research and clinical practice, as well as to identify potential targets and biomarkers for future strategies to reduce or even prevent PTLD.

## 2 Post-tuberculosis lung disease

Major efforts to combat TB have primarily focused on rapid diagnosis and effective treatment to break the chain of Mtb transmission, while the long-term consequences of the disease on survivors have largely been overlooked. Recently, however, it has become evident that in some cases, TB does not end with antimicrobial treatment and microbiological cure. It continues to chronically affect millions of people worldwide, causing significant physical, psychological, and economic burdens, along with a wide range of clinical, functional, and structural lung sequelae, ranging from mild to severe disorders (29).

Despite the recent increase in PTLD-related publications, its true epidemiological burden remains poorly understood (30). It is estimated that up to 50% of tuberculosis survivors experience some form of sequelae (31). Additionally, the mortality rate among these patients can be 3- to 6-fold higher compared to the general population (32–34). According to previous studies, the prevalence of PTLD ranges from 18% to 87% (8). This wide variation can be attributed to the different populations studied and the diverse criteria used to diagnose PTLD, which complicates the generalization of findings. Moreover, few studies have successfully correlated structural damage seen in chest imaging, functional impairment, respiratory symptoms, and quality of life.

One of the potential pathways leading to lung hyperinflammation and severe lung damage associated with PTLD is the deleterious neutrophil response and their antimicrobial factors (e.g. lysosomal enzymes, anti-microbial peptides, reactive oxygen species) released following Mtb phagocytosis. This neutrophil behavior contributes to the destruction of extracellular matrix components, preventing the resolution of inflammation, and inducing fibrosis (25, 27, 35, 36).

As a consequence of physical lung sequelae, persisting respiratory symptoms are common, including dyspnea, cough, wheezing, hemoptysis, and reduced exercise capacity, all of which may impair survivors’ routine activities. Because PTLD is a disease that can affect different compartments of the chest, diverse patterns of clinical presentation can occur even within a single patient. Obstructive lung disease and bronchiectasis are manifestations of airway disease. Fibrotic changes and cavitation are secondary to parenchymal destruction, as chronic pleural disease and pulmonary hypertension are conditions associated with post-TB pleural and vascular disease (30). Additionally, patients may experience recurrent bacterial, fungal, and mycobacterial infections.

As with clinical manifestations, the pulmonary function deficits observed in patients with PTLD can present in a variety of patterns (obstructive, restrictive, mixed and/or impaired gas exchange). In fact, approximately 10% of PTLD patients lose more than half of their lung function. Excessive inflammation, distortion and/or narrowing of the airways and destruction of the elastic and muscular components of the bronchial walls can ultimately lead to obstruction, resulting in a reduction in the ability to expel air from the lungs and a decrease in forced expiratory volume in one second (FEV_1_). A meta-analysis showed that previous TB was a risk factor for chronic obstructive pulmonary disease, with a pooled odds ratio of 3.05, independent of smoking and age (37). On the other hand, restriction can be attributed to fibrosis, stiffening of the lung parenchyma, fibrotic bands, bronchovascular distortion and pleural thickening, and can be detected in 24% of patients at the end of TB treatment (38). Although airflow obstruction in TB has received more comment, mixed patterns of airflow obstruction/restrictive ventilatory defects are very common as are impaired diffusing capacity and ventilation/perfusion mismatch measured by diffusion lung capacity (8). All of these issues are primarily due to cavitation, bronchiectasis, pleural thickening, fibrosis, and pulmonary hypertension resulting from severe lung damage caused by hyperinflammation during active disease (39, 40).

In practice, it is evident that PTLD patients bear the catastrophic financial burden of active disease, which has been prioritized within the ‘End TB’ agenda. Individuals cured of TB may face long-term socio-economic and psychological consequences, leading to ongoing economic, social, and psychological distress (41). However, data on these impacts of TB beyond treatment completion remain limited, and their effects on long-term well-being are poorly understood.

In 2023, the Second International Post-Tuberculosis Symposium identified six areas with high translational potential for a focused review of the literature:(1) tissue matrix destruction, including the role of matrix metalloproteinase (MMP) dysregulation and neutrophil activity, (2) fibroblasts and profibrotic activity, (3) granuloma fate and cell death pathways, (4) mycobacterial factors including pathogen burden, (5) animal models, and (6) the impact of key clinical risk factors including HIV, diabetes, smoking, malnutrition and alcohol (42, 43). In the next sections, we will discuss the role of neutrophils and their biomarkers in lung tissue damage during pulmonary TB, aiming to help fill the gaps in the TB and PTLD field.

## 3 The role of neutrophils during tuberculosis: “a double-edged sword”

The role of neutrophils in the immunopathogenesis of TB has been studied since 1909 (44). However, during the last century, macrophages and lymphocytes were considered the most relevant cells in the defense against Mtb, with macrophages serving as the frontline cells of the innate defense (45), and the lymphocytes responsible for containing the bacilli within the granuloma (46). Despite their protective role against Mtb, neutrophils have previously been questioned as “friend or foe?” (28), described as a “double-edged sword” (25), and termed as a “Trojan horse” (47), due to their greater contribution to the pulmonary pathology rather than only protecting the host during Mtb infection.

Neutrophils are innate immune cells involved in the process of killing Mtb during the early phases of infection. They are the first phagocytes to arrive from the circulation at the site of infection and attempt to eliminate invading pathogens (47). In the lungs, they accumulate in the airways of patients with active pulmonary TB, being the predominant phagocytic cells measured in sputum or bronchoalveolar lavage specimens during the disease (27, 48). This enhanced neutrophilic inflammation is pathogenic in TB, and high bacterial loads and lung pathology damage have been correlated in some animal models and human TB, creating a vicious cycle. On one hand, mycobacterial products and the pro-inflammatory environment of granulomas upregulate the neutrophil response; for instance bacterial products can drive neutrophil activation (49). On the other hand, neutrophils can promote Mtb growth by acting as a nutrient reservoir for fatty acids and cholesterol in the lung (24, 50). Furthermore, genetic polymorphisms in neutrophils may be associated with a dysregulated granulocytic influx into the infected tissue, promoting disease by creating a permissive intracellular niche for mycobacterial growth and persistence (22, 51).

The classical concept that neutrophils are merely fast responders with microbicidal effects attributed to the phagocytosis of bacilli has evolved, as new functions of these PMN cells have been identified. These include their capacity to release DNA traps in the extracellular environment aimed at capturing Mtb and the ability to produce a range of cytokines/chemokines which in turn may contribute to modulate the responsiveness of distant cells (52). In addition, neutrophils are known for their ability to interact with other cells, such as macrophages, dendritic cells, monocytes, and lymphocytes (22, 53).

To eliminate the bacilli, neutrophils display several functions, including chemotaxis, phagocytosis, generation of reactive oxygen metabolites, and activation of other immune cells (54). Phagocytosis is one of their fundamental functions, immediately followed by an oxidative burst, which is more intense than that observed in macrophages (55). During phagocytosis, Mtb is engulfed into neutrophil phagosomes, which rapidly fuse with intracellular granules to form phagolysosomes. This process leads to the generation of reactive oxygen species (ROS) and the release of proteolytic enzymes known as neutrophil serine peptidases (NSP) from the granules. These NSPs contribute to pathogen control, the formation of traps aimed at capturing the bacilli extracellularly, and subsequently apoptosis and autophagy (22, 56).

Meanwhile, it has been demonstrated that neutrophils also transport antigens from peripheral sites to lymph nodes and bone marrow, facilitating the generation of Th1, Th17, and CD8^+^ memory responses (57). The neutrophil-dendritic cells interaction enhances the maturation of dendritic cells, which are capable of internalizing neutrophils carrying Mtb and cross-presenting these antigens to T lymphocytes. As antigen-specific Th1 and Th17 cells emerge, a mixture of activated immune cells begin to cluster in the incipient granulomas (26). At this stage, the pathogen is confined in a dormant form, defined as latent TB infection (LTBI), and approximately 5-10% of patients with unstable immunity will develop active TB.

Under so-called normal conditions, neutrophils undergo spontaneous apoptosis after degranulation and are subsequently engulfed by macrophages, which leads to a reduction in inflammation. However, during TB infection, neutrophil apoptosis is delayed, and activated neutrophils may die by necrosis. This defective clearance of dead cells can perpetuate inflammation and result in progressive tissue damage (15, 23). As a systemic reflex of this neutrophil’s behavior, high blood leukocyte levels are observed during active TB, primarily due to neutrophilia (58–60). Furthermore, neutrophilia appears to be directly related to lung tissue damage during pulmonary TB, suggesting that this unresolved inflammation is associated with subsequent extensive fibrosis (61). Elevated peripheral blood neutrophil counts at baseline and during follow-up in patients with pulmonary TB have been linked to adverse microbiological and radiological outcomes (62–64) and are also associated with clinical outcomes, including being identified as a risk factor for death (58). In extrapulmonary TB, higher cerebrospinal fluid neutrophil counts and blood neutrophilia were also correlated with mortality in HIV-uninfected patients with TB meningitis (65). These higher levels of neutrophils are correlated to higher plasma and sputum levels of pro-inflammatory neutrophil-derived mediators and with severe lung damage at baseline and after treatment (66).

Blood neutrophils from active pulmonary TB patients present different phenotypes and functionality when exposed to Mtb-specific antigens before and after TB treatment. Higher frequencies of banded neutrophils, which usually circulate during some inflammation, and segmented neutrophils, systemically presenting on homeostatic conditions, at TB diagnosis were associated with more severe lung pathology. In addition, baseline granulocyte frequencies are increased in patients with poor recovery compared to those showing good recovery after TB treatment (67).

Neutrophil Lymphocyte Ratio (NLR) is another parameter studied to predict clinical and microbiological response. The decrease in white blood cell count, neutrophils and also in NLR have been observed in TB patients following TB treatment, indicating their potential as positive markers for disease follow up (68, 69). On the contrary, higher NLR levels during TB have been associated with delayed sputum smear conversion (70).

In the routine medical follow-up of TB patients, neutrophils are often neglected, even in the interpretation of a basic test like whole blood cell counts. Although nonspecific, baseline and follow-up peripheral blood neutrophil counts can serve as predictors of TB progression during treatment, particularly in countries with low socioeconomic status and a high TB burden, provided that alternative causes of neutrophilia are ruled out.

## 4 Neutrophil’s mechanisms of lung injury during pulmonary tuberculosis

**Figure 1:**
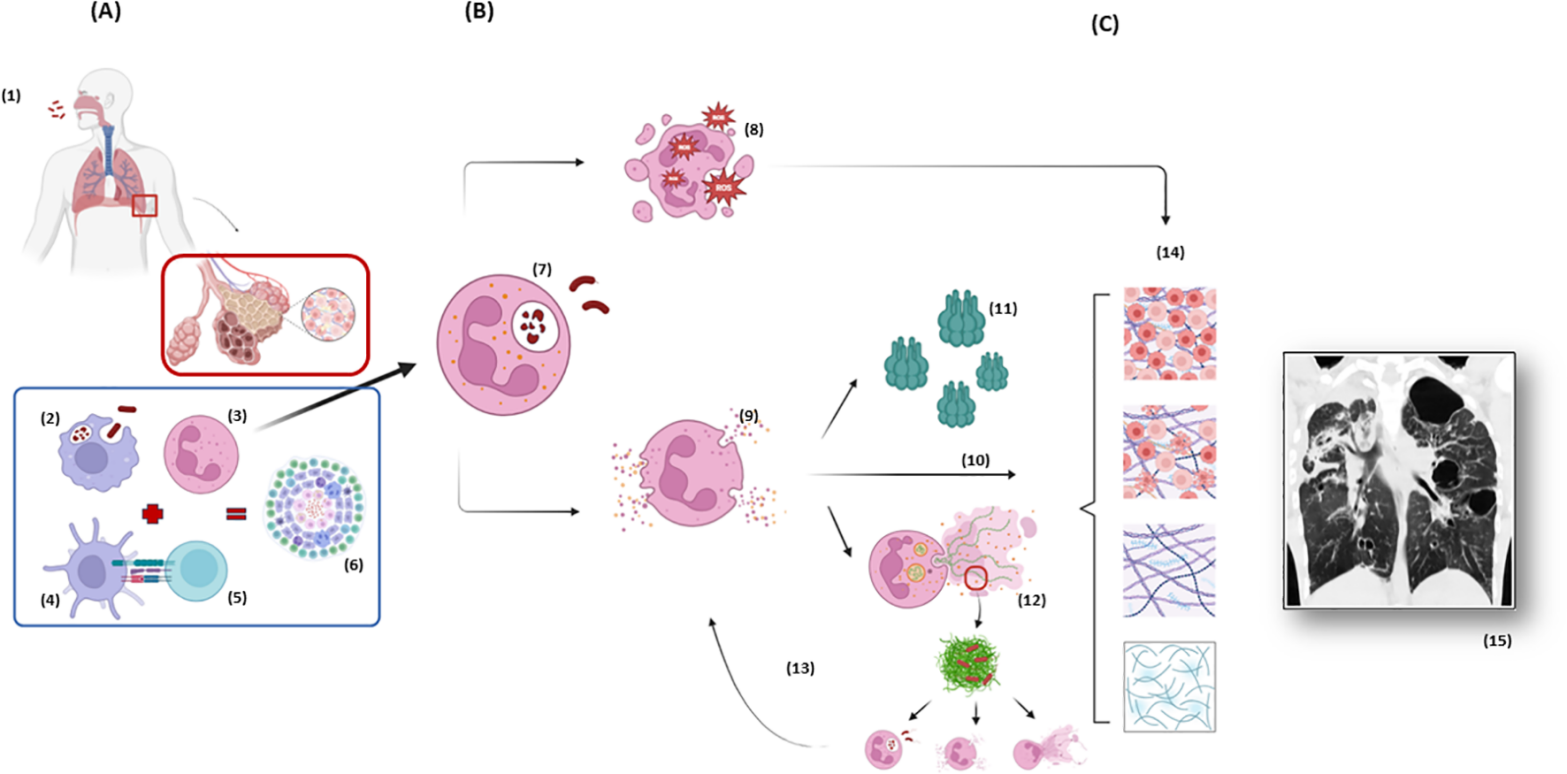
Neutrophil response during tuberculosis immunopathogenesis and the spotlight of neutrophil elastase. **(A)** After Mtb is inhaled and has reached the distal airways (1), the host response initiates through innate immunity with macrophages phagocytosis (2) and chemotaxis of neutrophils (3), the first cells to arrive at site of infection. Neutrophils participate in the crosstalk with dendritic cells (4) which works as an antigen presenting cell to T lymphocytes (5), starting the adaptative immune response which culminates in the containment of Mtb in the granulomas (6). **(B)** Based on a complex cascade of interactions between the pathogen, the environment and the host, an exacerbated neutrophil response can lead to damage of the ECM and tissue destruction. The phagocytosis of the mycobacteria by neutrophils (7) generates the formation of phagolysosomes and initiates an oxidative burst and the production of ROS (8) aiming to eliminate Mtb, but which can also damage lung tissue. The degranulation of NSPs (9) and the release of NE among other peptidases are the “core” of the cascade leading to hyperinflammation. NE itself has a direct effect on tissue digestion (10). NE has also the capacity of activating MMPs (11), endopeptidases with special ability of ECM destruction. Finally, NE presents an important role in the formation of NETs (12), which at first aims to catch and kill the mycobacteria but only succeeds in catching not killing the bacteria, leading to a positive feedback to hyperinflammation, as the extracellular Mtb attract more neutrophils amplifying the process (13). **(C)** All these pathways (10, 11, 12) are important components of a progressive ECM destruction (14), collagen deposition and exacerbated healing which end up in the conformation of radiological visible scars (15) and consequently clinical and respiratory function impairment which characterize Post-Tuberculosis Lung Disease. Figure from chest CT adapted from (71). Mtb, Mycobacterium tuberculosis; ECM, Extracellular Matrix; NSP, Neutrophil Serine Peptidases; ROS, Reactive Oxygen Species; NE, Neutrophil Elastase; MMPs, Matrix metalloproteinases; NET, Neutrophil Extracellular Trap; CT, Computed Tomography. Created in BioRender. dos santos, a. (2025) https://BioRender.com/r33n152 and https://BioRender.com/j08p350.

The association between neutrophils and lung damage during pulmonary TB is based on several pathways, including oxidative burst, the release of NSPs and neutrophil extracellular traps (NETs) and the production of lytic molecules [such as matrix metalloproteinases (MMPs), cathepsins, S100 proteins, cathelicidins, and β-defensins] (54). Some of these mechanisms are illustrated and summarized in **Figure 1** and **Table 1**, respectively, and will be discussed in detail below.

**Table 1:** Summary of the beneficial role of neutrophil products during tuberculosis immunopathogenesis, their respective mechanisms leading to hyperinflammation and lung tissue damage and the evidence of their correlation with post-tuberculosis lung disease.

Neutrophil product	Beneficial role during TB immunopathogenesis	Mechanisms of hyperinflammation and lung tissue damage during pulmonary TB	Evidence of correlation with lung tissue injury and PTLD
*Neutrophil Elastase*	NE is a serine protease found in the azurophilic granules of the neutrophils and released after phagocytosis of Mtb and formation of the phagolysosome. Together with MPO, cathepsin G, proteinase 3 and defensins, they are considered key antimicrobial components that play a critical role in eradicating the bacilli, protecting against mycobacterial replication and contributing to pathogen destruction.	• Enhance neutrophil migration by inducing the secretion of GM-CSF, IL-6, and IL-8 from epithelial cells. • Cleaves α1-antiprotease inhibitor to generate a fragment that is chemotactic for neutrophils. • Have as potential substrates: ECM proteins such as collagen, elastin, fibrin, fibronectin, the platelet IIb/IIIa receptor, and cadherins. • Promote endothelial and epithelial permeability and pulmonary edema. • Stimulate of collagen synthesis in fibroblasts. • Induce wound healing by releasing TGF-β from the ECM. • Activate MMPs and degrade their endogenous inhibitors, resulting in increased and sustained activity in the airways. • Are components of NETs that can increase protease activity and inflammation in the airways.	• Increased plasma levels of NE were found in active TB patients when compared to LTBI and to HC (59, 60, 72). • Decreased plasma levels of NE were measured during follow-up and TB treatment (72). • NE activity was associated with severe lung damage and pulmonary destruction as measured by lung imaging tests during active TB (64).
*Matrix Metalloproteinases*	MMPs are proteases regulated by RNA transcription and stored in various cells, such as neutrophils, to be released upon leukocyte activation. Only then, they are able to modulate chemokine gradients and regulate leukocyte recruitment to the sites of Mtb inflammation leading to cell migration and granuloma formation.	• Degrade ECM components. • Activate Il-1β, a potent fibrotic agent. • Cleave α1-antitrypsin, an anti-elastase protein, thereby enhancing NE activity. • Are upregulated by ESAT-6 of Mtb, disrupting the balance between them and their tissue inhibitors, thus also contributing to the ECM degradation.	• MMP – 1 levels in respiratory specimens were significantly elevated in TB patients when compared to HC, and the median levels of the MMP inhibitors were significantly lower in TB patients (73). • In respiratory specimens from pulmonary TB patients, MMP – 10 was elevated when compared to controls implicating it in TB-associated tissue destruction (74). • Increased levels of MMP – 1 and MMP – 3 in the induced sputum of pulmonary TB patients were independently associated with higher TB clinical severity scores (75). • Plasma levels of MMP – 1, − 2, − 3, − 9 and − 12 were significantly higher in active TB patients with cavitary and/or bilateral disease at baseline (76). • Plasma levels of MMP – 1, − 2, − 3 and − 8 showed a significant positive association with bacterial load measured by smear microscopy (76). • Plasma levels of MMP – 8 were associated with mortality in a cohort of advanced HIV-positive individuals (77).
*Neutrophil Extracellular Traps*	NETs are web-like structures composed of chromatin and antimicrobial proteins released by neutrophils to capture and destroy pathogens that are trapped within their networks. The DNA strands within NETs act as a physical barrier that immobilizes the Mtb, while the antimicrobial proteins associated with NETs, such as histones, NE, and MPO, use their microbicidal properties to directly kill pathogens. NETs are also capable of modulating immune responses and activating other immune cells, such as macrophages and dendritic cells, to enhance their antimicrobial activities.	• NETs trap mycobacteria *in vitro* but are unable to kill them. The presence of the live pathogen in the trap also stimulates unwanted immune responses and triggers tissue damage. • Mtb has been shown to trigger NE release of NETs in plasma and in sputum samples from infected individuals, resulting in higher concentrations of NE in TB patients when compared to uninfected controls.	• Levels of NET markers were higher even after TB treatment initiation in those who did not show radiologic improvement in the second month of treatment (63). • Increased plasma/serum levels of NET markers were associated with severe lung damage during active TB (63, 72, 78). • Measurements of NET components showed higher levels in pulmonary TB patients when compared to healthy controls and these levels were significantly higher in cases of TB relapse and in patients with more lung destruction (64).

TB, Tuberculosis; PTLD, Post-Tuberculosis Lung Disease; Mtb, *Mycobacterium tuberculosis*; MPO, myeloperoxidase; GM-CSF, Granulocyte-macrophage colony-stimulating factor; Il, Interleukin; ECM, extracellular matrix; TGF-β, Transforming Growth Factor-Beta; MMP, Matrix Metalloproteinase; NET, Neutrophil Extracellular Trap; N, Neutrophil Elastase; LTBI, Latent Tuberculosis Infection; HC, Healthy Controls; ESAT-6, Early secretory antigen target-6; HIV, Human Immunodeficiency Virus.

### 4.1 Oxidative burst

Reactive oxygen species are generated as by-products of cellular metabolism, primarily in the mitochondria. When the cellular production of ROS exceeds its antioxidant capacity, damage to cellular macromolecules such as lipids, proteins, and DNA may occur. This state of “oxidative stress,” or oxidative burst, is thought to contribute to the pathogenesis of several human diseases (79), including TB (80). Although ROS can damage host cells, they also play a crucial role in killing mycobacteria and other invading pathogens within the host. It is important to note that the survival of Mtb is highly dependent on the levels of ROS produced by the host immune cells. If these levels are insufficient to counteract the antioxidant systems of Mtb, the pathogen can continue to survive and replicate (81). Thus, the balance of oxidative responses needs to be studied and addressed, as it is a critical component in the battle against mycobacteria. Several established antituberculosis antibiotics are administered in an inactive form and are subsequently transformed into their active form by components of the oxidative burst responses from both the host and the pathogen (80).

Using other disease models, oxidative stress has been found to play an important role in pulmonary fibrosis by inducing lung collagen deposition (82). In a review of interstitial lung diseases (83), oxidative imbalance (i.e., increased ROS or decreased antioxidants) can directly damage the epithelium and lead to degradation of the extracellular matrix in the lungs. This results in irreversible injury that causes scar tissue deposition and a further loss of antioxidant enzymes. Additionally, ROS and reactive nitrogen species (RNS) can contribute to the pathogenesis of pulmonary fibrosis by altering the expression of other fibrotic mediators, such as transforming growth factor-β (TGF-β), proteases, and antiproteases. An association between greater declines in TGF-β levels during early TB treatment and better lung function post-treatment was previously demonstrated (84). The same study also found that patients with the worst lung function post-treatment parameters had a paradoxical increase in TGF-β levels during the first 2 months of treatment.

Measuring oxidative burst is challenging because these techniques are often laborious, primarily due to the need to isolate neutrophils. It is essential to develop efficient, simple, and highly reproducible techniques to quantify ROS generation by PMN cells for use as predictors of host defense or injury. The development of a method to measure oxidative burst in neutrophils suspended in heparinized whole blood could facilitate routine clinical application (85). This technique employs flow cytometry and has been considered a simple and reliable method for assessing polymorphonuclear function in trauma patients (86).

### 4.2 Neutrophil serine peptidases and the spotlight of neutrophil elastase

Upon phagocytosis of pathogens, neutrophils undergo rapid degranulation, during which cytoplasmic granules fuse with the phagocytic vacuole, releasing hydrolases and bactericidal proteins (56). The degranulation process is tightly regulated and leads to the secretion of four main types of enzymes known as neutrophil serine peptidases: a) Primary (Azurophilic) Granules: These granules contain key antimicrobial components, including neutrophil elastase (NE), myeloperoxidase (MPO), cathepsin G, proteinase 3, and defensins, which play critical roles in the eradication of Mtb; b) Secondary (Specific) Granules: Rich in lactoferrin, these granules sequester iron, copper, and various proteins, thereby inhibiting the growth of mycobacteria; c) Tertiary (Gelatinase) Granules: These granules contain gelatinases, such as MMPs, which facilitate a variety of functions, including the degradation of the extracellular matrix and the activation of IL-1β, a potent fibrosing agent; 4) Secretory Granules: These granules primarily contain albumin and various cytokines, contributing to the modulation of the inflammatory response (21).

Neutrophil elastase, cathepsin G, and proteinase 3 play crucial roles in combatting Mtb. Their controlled activation and release are essential for host defense, and while they effectively protect against mycobacterial replication and contribute to pathogen destruction in conjunction with ROS, their elevated levels can also cause significant damage to normal tissues (87). As demonstrated in animal models, neutrophil-derived elastase and cathepsin G are vital in slowing pathogen replication during the early stages of antimycobacterial responses (88). However, this protective function may be compromised by their potential deleterious effects. Evidence from animal studies indicates that mice deficient in these three neutrophil serine peptidases are substantially protected from lung tissue destruction following prolonged exposure to cigarette smoke. Furthermore, the activity of NSPs is associated with an increased release of tissue-destructive proteases, such as MMPs – 9 and – 12 (89).

Clinical and experimental studies have elucidated the role of NE in various lung diseases and its potential to promote pathological extracellular matrix accumulation (90). Neutrophil elastase is believed to modulate inflammatory regulation through both direct and indirect mechanisms (91). Directly, NE, when expressed on the cell surface, can stimulate collagen synthesis in fibroblasts, contributing to extracellular matrix deposition and subsequent fibrosis (92, 93). Indirectly, NE triggers the release of extracellular traps and exosomes, which can enhance protease activity and inflammation within the airways. Additionally, NE interacts with other proteases, such as MMPs, which are not only involved in extracellular matrix degradation but also cleave α1-antitrypsin, an anti-elastase protein, thereby amplifying NE activity (94, 95). Moreover, NE may play a role in regulating wound healing through the release of transforming growth factor-beta (TGF-β) from the extracellular matrix. Another concerning property of NE is its cytotoxicity toward endothelial cells, which can lead to increased vascular permeability and alveolar edema when present in excess (96). The influence of NE on lung damage during TB immunopathogenesis have been studied in *in vitro* models, using human neutrophils stimulated by Mtb. Hypoxia induced during TB infection influences the up-regulating pathways that increase NE and MMPs secretion that are implicated in matrix destruction and lung damage (97).

*In vivo*, plasma levels of human NE were increased in active TB when compared to LTBI patients and to healthy controls. In addition, elastase decreased during follow-up and treatment (59, 60, 72). Besides that, NE was also associated to severe lung damage and pulmonary destruction measured by pulmonary imaging during active TB (64).

We dare say that NE can be considered the core of the immunopathology leading to lung damage in pulmonary TB, as it is directly related to tissue degradation (96), in addition to being part of the extracellular traps involved in pulmonary hyperinflammation (60) and for its role in the release of MMPs, classically known to be detrimental to extracellular matrix components (98). Based on what is known to date, NE has been identified as a promising biomarker for the early diagnosis of lung inflammation and, together with radiological imaging, could predict those at higher risk of lung damage and consequently PTLD.

Recent studies have shown an increasing interest in developing methods for measuring NE activity (99, 100). Several assays have been developed to measure active NE in sputum samples and different principles for activity determination have been employed (100). Commercial kits for NE measurements suitable for the analysis of most cell culture supernatant, sputum, serum, and plasma samples are available using Enzyme Linked Immunosorbent Assay (ELISA) techniques. Although usually used for research purposes, in the future, they could be applied for clinical assistance as biomarkers for TB diagnosis and as predictors for PTLD.

### 4.3 Matrix metalloproteinases

Matrix metalloproteinases are a family of 25 potent proteases that can modulate chemokine gradients and regulate leukocyte recruitment to sites of inflammation caused by *M. tuberculosis* (101). However, they can also degrade extracellular matrix components and are probably central to TB-associated lung injury based on different stages of lung remodeling during the disease (98). These proteases are regulated by RNA transcription and can be stored in different cells and only after activated they can be released. Pro-MMP – 8, pro-MMP – 9 and pro-MMP – 25 are packed into peroxidase-negative granules within neutrophils to be released upon leukocyte activation. Peroxidase-positive azurophil granules contain both activators of MMPs, such as NE and ROS-generating enzymes, and inactivators of MMPs, such as thrombospondin (102, 103).

Studies indicate that NE cleaves inhibitory protein residue in several MMPs, including MMP – 2 and MMP – 9, and can also degrade their endogenous inhibitors, or TIMPs. These data suggest that NE may increase MMPs activation and reduce their inhibition, leading to its increased and persistent activity in the airways and thus airway remodeling and lung damage (102). Besides that, Mtb infection leads to upregulation of MMPs and causes disturbance in the balance between MMPs and their tissue inhibitors, thus also contributing to the extracellular matrix degradation (98).

In fact, several studies with animals and humans published *in vitro* or *in vivo* evidence MMPs participation in lung damage during pulmonary TB (104). In the transgenic mice, Mtb increased MMP – 1 expression, resulting in alveolar destruction in lung granulomas and significantly greater collagen breakdown (73). In another study, MMP activity was upregulated in a rabbit model and the expression of MMPs – 1, – 2, – 3, – 9, – 12, – 13, and – 14 was associated with destructive pathology in the lung of the animals (105).

An *in human* study, using plasma from pulmonary TB patients showed a decrease in concentrations of MMPs – 1, – 8 and – 10 during treatment. In addition, increased plasma levels of MMP – 8 was associated with mortality in a cohort of advanced HIV positive hospitalized individuals, suggesting that MMP upregulation and matrix turnover are features of TB disease severity (77). Additionally, circulating levels of MMPs – 1, − 2, − 3, − 7, − 10 and − 12 were significantly higher in patients with TB and diabetes mellitus compared to both TB and healthy controls. Moreover, the levels of MMPs – 1, − 2, − 3, − 9 and − 12 were significantly higher in active TB individuals with cavitary and/or bilateral disease at baseline. Also, levels of MMPs – 1, − 2, − 3 and − 8 exhibited a significant positive relationship with bacterial load measured by smear microscopy (76).

In respiratory specimens of pulmonary TB patients, MMP – 10 was significantly increased when compared to controls implicating it in TB-associated tissue destruction. This increase was supposed to be partly driven by early secretory antigen target-6 (ESAT-6), a protein secreted by the mycobacteria, responsible for its escape from phagosome to cytoplasm of cells (74). The measurements of MMPs – 1, − 2, − 3, – 8, − 9 were also at higher levels in the induced sputum of pulmonary TB patients when compared to controls. Particularly MMPs – 1 and – 3 levels were independently associated with higher TB clinical severity scores and MMP – 2, – 8, and – 9 were increased at TB diagnosis in patients who remain sputum culture positive at two weeks, suggesting a capacity to predict unfavorable outcome (75). Finally, MMP – 1 concentration in respiratory specimens was significantly increased in TB patients when compared to healthy controls, contrasting with the reduced levels of the their inhibitors (TIMP-1 and TIMP-2) (73).

Among other technologies (106), the measurement of MMPs can be performed by using ELISA techniques with commercial kits available for research purposes.

### 4.4 Neutrophil extracellular traps

Neutrophil extracellular traps are web-like structures composed of chromatin and antimicrobial proteins released by neutrophils (107–109). Their primary function is to capture and destroy pathogens that are trapped in their networks. (110). The DNA strands within NETs act as a physical barrier, immobilizing microorganisms, and at the same time, the antimicrobial proteins associated with NETs, such as histones, NE, and MPO, use their microbicidal properties to directly kill pathogens (111). Human NE and MPO are essential for NET formation and both these enzymes are found within primary granules of resting neutrophils. While NE cleaves histones to unveil DNA, MPO assists NE to translocate to the nucleus during the process of NET formation (112). In addition to their main objective, NETs are also capable of modulating immune responses and activating other immune cells, such as macrophages and dendritic cells, to enhance their antimicrobial activities (109).

Despite its protective role, NETs can be potentially harmful to the host because of its toxic components which can harm healthy cells and contribute to chronic inflammation (113–115). In TB immunopathogenesis, Mtb is reported to induce the formation of NETs, which trap mycobacteria *in vitro* but are unable to kill them (116, 117). The presence of the alive pathogens in the trap also stimulates unwanted immune reactions and trigger tissue injury (27), i.e. NETs contain the bacilli, but the ineffective clearance of the bacteria poses pathological consequences to the lungs.

The effect of Mtb on NET induction might be mediated by the virulent factor ESAT-6 (118), which is also secreted in large quantities in the extracellular space and therefore can interact with immune cells to stimulate them and facilitate the maintenance of chronic inflammation in the lungs of TB patients (119). Mtb has been shown to trigger NE release of NETs in plasma and in sputum specimens from infected subjects, leading to higher concentrations of NE in TB patients when compared to uninfected controls (60, 117, 120).

Increased plasma/serum levels of NET markers, including extracellular DNA, nucleosome, NE, MPO or citrullinated histone H3 (CitH3) were associated with severe lung damage during active TB (63, 72, 78). Levels of NET markers were also higher even after TB treatment initiation in those who did not improve in the second month of treatment (63). NET components (cell-free DNA, NE-DNA complexes, MPO-DNA complexes) and NE activity measurements present at higher levels in pulmonary TB patients when compared to healthy controls. In addition, these levels are significantly increased in cases of TB relapse (*versus* first TB episode) and in patients with more lung destruction (64).

In addition to qualitatively visualizing NETs, they can also be quantified (121). The most common methods include quantification of the area covered by extracellular DNA after staining (122), fluorescence spectroscopy of the culture media after adding DNA-binding dyes, or a modified ELISA against Cit3, MPO-DNA or NE-DNA complexes characteristic of NETs (123). Despite being less laborious to measure NETs nowadays, these tests are only used for research purposes, and they can be very useful in research of host directed therapies against TB.

## 5 Neutrophils and TB: from bench to bedside

Recent research has illuminated the role of neutrophils in the immune response to TB, revealing their potential impact on disease outcomes. This review aims to explore how neutrophil biomarkers could be leveraged in clinical practice, particularly for monitoring pulmonary TB patients, identifying those at greater risk for developing PTLD, and used as targets for host directed therapies (HDT).

One common aspect among the neutrophil products analyzed in this review (NE, MMPs and NETs) is the laboratory technique used for their measurement. The ELISAs are one of the primary methods for detecting and quantifying various biomarkers, including those mentioned here. This technique is relatively straightforward to perform, widely utilized in clinical laboratories, and available with commercial kits that offer a favorable cost-benefit ratio (124).

Although NE monitoring is not used as a laboratory measurement for routine purposes, it has been considered an important therapeutic target, with ongoing trials exploring its inhibition in both oncology and pulmonology, such as acute lung injury, severe acute distress syndrome and pneumonia, with contrasting results (125). Recently, brensocatib (INS1007), a potent, selective, competitive and reversible dipeptidyl peptidase I (DPP-1, also known as cathepsin C) inhibitor was shown to inhibit the formation of all 3 active NSPs (NE, proteinase-3 and cathepsin-G) in maturing neutrophils *in vitro* and *in vivo*. It was developed for non-cystic fibrosis bronchiectasis treatment, aiming to reduce pulmonary exacerbations along with prevention of disease progression, to maintain or improve lung function, and to improve the symptoms and quality of life. The drug has passed through phase 1, phase 2 (WILLOW trial) (126) and phase 3 (ASPEN trial) (results not published yet) studies with promising results. The WILLOW trial demonstrated that treatment with brensocatib, significantly prolonged the time to the first exacerbation in patients with bronchiectasis compared to placebo, while also decreasing sputum NE activity without raising safety concerns (126). Despite this promising evidence of NE’s role in lung damage in pulmonary TB there have been no experimental or clinical trials using NE inhibitors as HTD for TB to date, as noted on ClinicalTrials.org. This represents an important gap in research, suggesting that innovative therapeutic strategies targeting neutrophil activity could offer new avenues for improving patient outcomes in TB management.

Sivelestat is another neutrophil elastase inhibitor under investigation for several pulmonary diseases, including chronic obstructive pulmonary disease, pulmonary fibrosis, and COVID-19 (127). Just like brensocatib, no trials are registered to study its use as an HDT in TB during the construction of this review.

Some scientific publications already discussed MMPs inhibitors as an alternative approach in managing lung tissue injury and its long-term consequences of pulmonary TB (98, 128, 129). A phase II double-blind and randomized controlled trial investigated doxycycline, a licensed broad-spectrum MMP inhibitor, in patients with pulmonary TB. The results showed that doxycycline significantly reduced sputum measurements of MMP – 1, – 8, – 9, – 12 and – 13, suppressed type I collagen and elastin destruction, reduced pulmonary cavity volume without altering sputum mycobacterial loads, and was safe (128). Three other studies are registered on ClinicalTrials.org regarding the use of doxycycline for TB as HDTs (NCT05473520, NCT06477185 and NCT06446245).

Despite the recent recognition of potential targets for host-directed therapies in TB, we don’t yet have any recommendations for specific therapeutic strategies in this direction, as no large clinical trials have been conducted that aim to reduce inflammation while the antimicrobials act to kill the pathogen itself. However, we understand that studies in the pathogenesis of PTLD is the first step to open opportunities for future research and implementation of HDT in TB.

## 6 Conclusion

The interplay between the pathogen, the host and the environment leads to lung damage caused by Mtb, resulting in the radiological, functional and clinical manifestations of PTLD. To alleviate the burden on TB survivors, strategies must focus on reducing pulmonary TB sequelae. This includes strengthening efforts to control TB transmission through early detection, effective short-term treatments, and preventive measures targeting social determinants of health. In addition, HDTs that mitigate lung damage are essential for reducing the long-term consequences of TB and identifying factors that contribute to lung hyperinflammation is crucial for developing these interventions.

In this narrative review, we have emphasized the dual role of neutrophils in active TB, highlighting their essential functions in both the phagocytosis and Mtb killing and the hyperinflammatory responses that contribute to tissue damage. The inflammatory cascade induced by NE not only directly damages the extracellular matrix but also promotes the formation of NETs, which further amplify the inflammatory response and activate MMPs. These processes collectively lead to the destruction of lung parenchyma during active TB, providing critical insights into potential leverage points for future HDTs. Targeting these mechanisms may help mitigate lung damage and improve outcomes for TB survivors, improving long-term survival with better quality of life, and underscoring the need for ongoing research into neutrophil biology and its implications for TB treatment strategies.
